# OA18 Small vessel and large vessel vasculitis due to rheumatoid vasculitis: a case of mononeuritis multiplex with pulseless foot leading to amputation

**DOI:** 10.1093/rap/rkad070.018

**Published:** 2023-09-27

**Authors:** Chamith Rosa, Mousfata Hosni, Siwalik Banerjee

**Affiliations:** University Hospitals Coventry and Warwickshire NHS Trust, Coventry, United Kingdom; University Hospitals Coventry and Warwickshire NHS Trust, Coventry, United Kingdom; University Hospitals Coventry and Warwickshire NHS Trust, Coventry, United Kingdom

## Abstract

**Introduction:**

Rheumatoid vasculitis occurs in patients with longstanding severe seropositive rheumatoid arthritis. The incidence has reduced drastically with availability of more effective medication. Rheumatoid vasculitis primarily affects small and medium vessels, and rarely large vessels as well.

**Case description:**

This 77-year-old lady with deforming seropositive rheumatoid arthritis was stable on methotrexate under the care of multiple Trusts for the last 30 years. Methotrexate had been stopped a year ago due to pancytopenia and she was started on prednisolone and hydroxychloroquine. While on these medications she flared with arthralgia and had difficulty in walking attributed to ankle synovitis. She was started on adalimumab, after 3 doses of which she developed widespread skin psoriasis leading to discontinuation. Subsequently, she was admitted to our Trust with left foot gangrene and was referred to rheumatology. Further questioning revealed that numbness in both lower limbs, inability to walk and frequent falls had started following the cessation of methotrexate.

On examination, there was bilateral foot drop, worse on the right side. The left foot was gangrenous with diminished pulses in dorsalis pedis and posterior tibial arteries with preserved all other pulses. There was no vasculitic rash or nail fold infarcts. Psoriatic rash had resolved and there was no active synovitis in the joints. Nerve conduction test revealed severe ongoing axonal degenerative type sensory and motor polyneuropathy in the lower limbs without any upper limb involvement. CT angiogram showed multifocal stenoses, more so in the left common iliac, external iliac and superficial femoral artery. There were no conventional vascular risk factors such as hypertension, diabetes, dyslipidaemia or smoking, and there was no past history of cardiovascular disease. PET-CT scan did not reveal any features of vasculitis or aortitis. The vasculitis screening was negative including cryoglobulins.

Initially she was on IV antibiotics for left foot cellulitis, hence she was managed with steroids without any potent immunosuppressive agents. Subsequently, she underwent left sided below knee amputation. After the wound had healed, she was started on IV rituximab with low-dose methotrexate and steroid taper.

**Discussion:**

Although rare, rheumatoid vasculitis can cause significant morbidity and complications. Rheumatoid vasculitis tends to occur in patients with so-called ‘burnt-out’ RA and strongly associated with seropositivity. While it can affect many organ systems, skin and the peripheral nervous system are most frequently affected.

Cutaneous vasculitis occurs in around 90% of patients and is the most common clinical manifestation. This includes parable purpura, leg ulcers and nailfold infarcts. It may lead to digital ischaemia, necrosis and gangrene if large vessels are involved. Another common manifestation is a vasculitic neuropathy due to involvement of the vaso nervorum. Around 40% have a sensory neuropathy and 20% have a mixed sensory motor neuropathy like the index patient.

Ocular manifestations such as scleritis have been described and may be the first indication of rheumatoid vasculitis. Aortitis in rheumatoid vasculitis may present with aortic insufficiency and aneurysm formation. Pericarditis has also been described as a possible disease manifestation in patients with rheumatoid vasculitis. Our patient did not have any of these clinical features and the PET-CT scan was unremarkable, which could have been due to the steroids she was on prior to the scan. Clinically, there was pulseless gangrene of the left foot with CT angiogram showing multiple stenosis suggesting large vessel involvement in the absence of conventional vascular risk factors.

Renal involvement in rheumatoid vasculitis is very rare. PAN like renal involvement and glomerulonephritis has been described. Our patient did not have any haematuria or any significant proteinuria, and imaging did not reveal any renovascular aneurysms.

Isolated nailfold infarcts do not require any systemic treatment. Systemic involvement with rheumatoid vasculitis requires aggressive treatment with high-dose steroids with cyclophosphamide or rituximab. Our patient had neuropathy and gangrene of the left foot, hence she was started on rituximab accordingly.

**Key learning points:**

Rheumatoid vasculitis is a rare but serious complication which can present with a vasculitic neuropathy/mononeuritis multiplex.

Rarely, it can cause ischemia and gangrene.

If there is systemic involvement, aggressive therapy with steroids, cyclophosphamide or rituximab is needed.

